# A Unique Patient Presentation of Disseminated Strongyloidiasis: Unraveling the Clinical Complexity of an Immunocompromised Patient

**DOI:** 10.7759/cureus.46408

**Published:** 2023-10-03

**Authors:** Yulia Shtanko, Sophia Perez, Lorena Del Pilar Bonilla

**Affiliations:** 1 Medical School, Herbert Wertheim College of Medicine, Miami, USA; 2 Internal Medicine, Baptist Health South Florida, Miami, USA; 3 Translational Medicine, Herbert Wertheim College of Medicine, Miami, USA

**Keywords:** covid-19, siadh, corticosteroids, eosinophilia, strongyloides stercoralis hyperinfection syndrome, strongyloidiasis

## Abstract

Strongyloidiasis is a rare parasitic disease that can remain dormant and asymptomatic in many individuals. However, in cases of immunosuppression, the motility rate of the *Strongyloides* parasite increases significantly. This case study presents a unique clinical scenario involving an 88-year-old Hispanic male with a disseminated *Strongyloides*infection. The patient's medical history includes coronary artery disease, a history of percutaneous coronary intervention, heart failure with reduced ejection fraction and subsequent recovery of left ventricular function, hypertension, dyslipidemia, mantle cell lymphoma being treated with rituximab every two months since 2019, and chronic anemia. This case emphasizes the importance for physicians to consider strongyloidiasis when faced with a diverse range of symptoms, including syndrome of inappropriate antidiuretic hormone secretion (SIADH), rash, gastrointestinal upset, urinary retention, chronic anemia, and chronic eosinophilia, as these manifestations may share a common origin.

## Introduction

Strongyloidiasis, most commonly caused by the nematode *Strongyloides stercoralis*, is an intestinal parasitic infection prevalent in tropical and subtropical regions worldwide [1,2]. While typically asymptomatic or exhibiting mild gastrointestinal symptoms, strongyloidiasis can also become a life-threatening condition in immunocompromised individuals. 

The life cycle of the parasite, *Strongyloides stercoralis*, involves direct, indirect, and autoinfection cycles, with larvae penetrating the skin, migrating to the lungs and gastrointestinal system, and producing eggs [1-3]. The parasite can be found in certain soil-grown vegetables and tap water [1,2]. Environmental risk factors include soil contact, poor sanitation, and proximity to farm animals. Children and individuals with low socioeconomic status are particularly vulnerable. 

Patients afflicted with strongyloidiasis can present with rashes, gastrointestinal and respiratory manifestations, and constitutional symptoms. Blood work can reveal peripheral eosinophilia and hyponatremia. In immunocompromised individuals, severe complications can arise, such as hyperinfection syndrome [1,2]. Hyperinfection syndrome is characterized by an explosive increase in the production of infective larvae, leading to a higher worm burden within the host [1,4]. This sudden escalation can cause complications, such as intestinal obstruction, peritonitis, gastrointestinal bleeding, pneumonitis, alveolar hemorrhage, respiratory failure, and sepsis [1,2]. Disseminated strongyloidiasis, involving the parasite's spread outside its ordinary life cycle, can result in bloodborne infection, weight loss, meningitis, encephalitis, and syndrome of inappropriate antidiuretic hormone secretion (SIADH) [1]. 

Various techniques are utilized to identify *Strongyloides *infection, including stool examination for larval forms, blood agar plate culture, serologic testing using enzyme-linked immunosorbent assay (ELISA) or indirect immuno-fluorescent antibody test (IFAT), and microscopic visualization of larvae in body fluids [1]. Real-time polymerase chain reaction (PCR) can also be used. Treatment of strongyloidiasis includes ivermectin, albendazole, or thiabendazole medication [1,5]. However, repeat dosing and continued treatment may be necessary. For patients with disseminated strongyloidiasis, it is recommended to give 200 μg/kg·d of ivermectin until stool or sputum exams are clear of infection; however, the duration of treatment for disseminated infection is not yet fully understood.

## Case presentation

An 88-year-old Hispanic male with a past medical history of mantle cell lymphoma, previous pulmonary embolism/protamine gene mutation, essential hypertension, diastolic heart failure, coronary artery disease, chronic anemia, and benign prostatic hyperplasia presented to the emergency department (ED) with altered mental status (AMS) according to his family.

He had a recent admission for COVID-19 pneumonia from June 12, 2023 to June 17, 2023 and was discharged home with corticosteroids. He presented again to the ED the following day with AMS and urinary retention for which a foley catheter was placed upon admission. Initial laboratory tests showed neutrophilic leukocytosis, hypochloremia, and positive *Strongyloides* IgG antibody from a previous admission (Table 1). Physical exam findings on admission were significant for signs of AMS, decreased breath sounds bilaterally, and livedo reticularis on his abdomen (Table 2). In addition, his urinalysis was unremarkable, aside from an elevated urine pH (Table 3).

**Table 1:** Laboratory findings

Admission laboratory	Results
Hemoglobin	13.1 g/dL
Hematocrit	38.7 %
WBC	18.3 K/uL *high*
Sodium on blood	135 mmol/L
Potassium on blood	4 mmol/L
Chloride on blood	96 mmol/L *low*
Strongyloides IgG antibodies	Positive
Four days after admission	
Sodium on blood	122 mmol/L *low*
Eosinophils	23 *high*

**Table 2:** Physical exam findings on admission

System	Findings
General	Obese, noncooperative with interview, aphasic
Head, eyes, ears, nose, throat (HEENT)	Normocephalic, moist mucous membranes
Neck	Supple, without masses, no elevated jugular venous pressure (JVP), trachea midline
Pulmonary	Decreased breath sounds bilaterally without wheezes, rales, or rhonchi, no accessory muscle use
Cardiac	Regular rate and rhythm, S1, S2. No murmurs, rubs, gallops. No peripheral edema
Abdomen	Distended, tympanic, diffusely tender to palpation
Lymphatic	No cervical adenopathy
Musculoskeletal	No cyanosis, clubbing, or edema
Skin	Livedo reticularis noticed on abdomen
Neuro	Noncooperative with neurological examination, aphasic

**Table 3:** Urinalysis on admission

Urinalysis	Results
Color	Yellow
Appearance	Clear
Glucose	Negative
Bilirubin	Negative
Acetone (ketones)	Negative
Specific gravity	1.013
Blood	Negative
pH in urine	8.5 *high*
Protein	Negative
Nitrite	Negative
Leukocyte esterase	Negative

**Figure 1:**
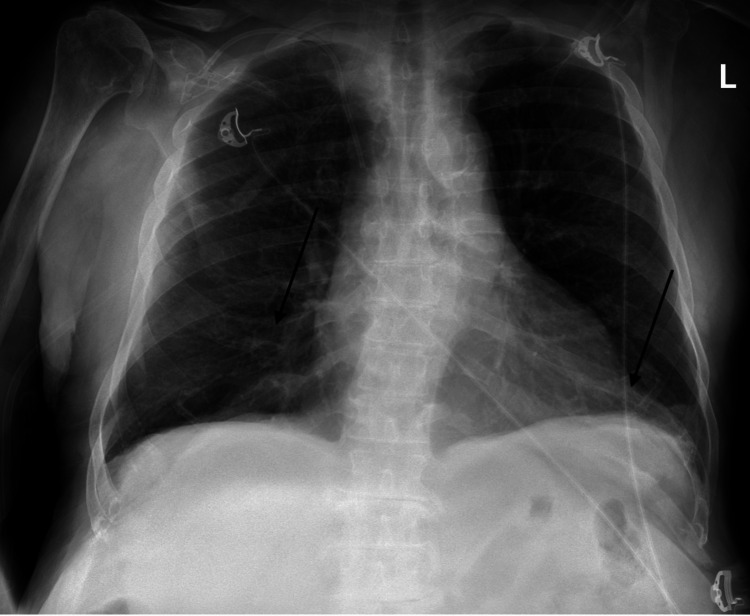
Chest X-ray on admission The arrows indicate bilateral pulmonary edema. There is no evidence of substantial pleural effusions.

**Figure 2:**
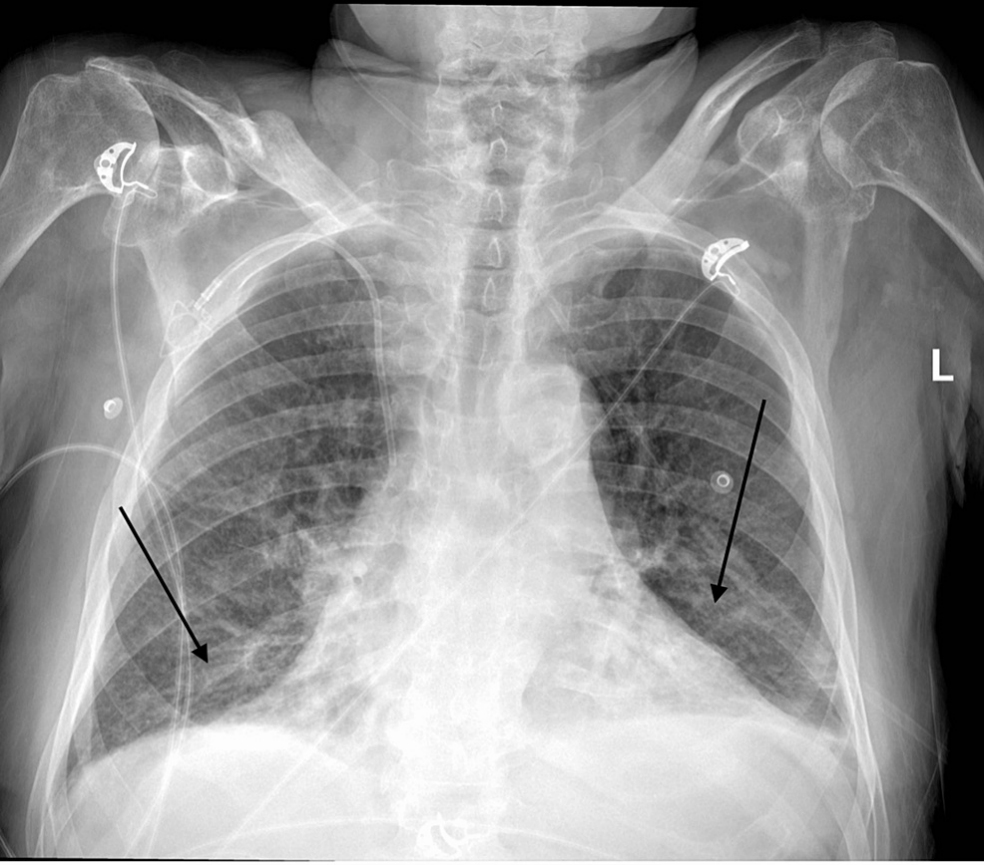
Follow-up chest X-ray The arrows on the follow-up chest X-ray indicate newly developed bilateral, bibasilar, parenchymal opacities.

**Figure 3:**
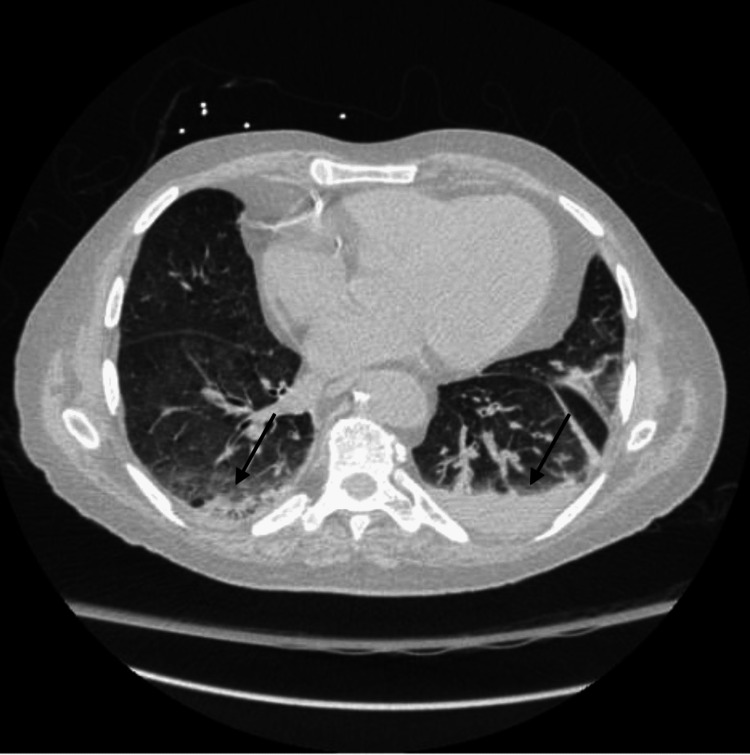
Chest CT scan Chest CT scan demonstrates bilateral pleural effusions and pulmonary infiltrates, as indicated by the arrows.

Initial chest X-ray at admission revealed pulmonary edema (Figure 1). Follow-up chest X-ray, six days later, showed persistent bibasilar parenchymal opacities suggesting edema or infiltrates (Figure 2). In addition, follow-up chest CT, also six days later, demonstrated small bilateral pleural effusions with areas of atelectasis in bilateral lower lobes and ground-glass attenuation infiltrates (Figure 3). He was admitted to the inpatient medical unit for further monitoring. 

A thorough review of his charts showed eosinophilia for a minimum of two years and a history of chronic anemia. Further laboratory workup taken after inpatient admission showed hyponatremia, most likely secondary to the development of SIADH, due to the patient's euvolemic status (Table 1). He also complained of reduced appetite, diarrhea, and a mild rash preceded by fever that subsided after acetaminophen administration.

After admission, an attempt was made to determine the cause of his urinary retention. It was discovered that he was taking oxybutynin for urinary incontinence, which could have induced his symptoms. In addition, his serology was positive for IgG strongyloidiasis and peripheral eosinophilia (Table 1). The infectious disease physician on call ordered a two-day course of ivermectin treatment, with a repeat regimen in two weeks.

It is important to note that his chest CT demonstrated infiltrates (Figure 3). While this may be due to superimposed bacterial pneumonia secondary to infection with COVID-19, there has also been evidence of infiltrates with reticulonodular opacities and hemoptysis seen in patients with disseminated strongyloidiasis [6].

This patient likely had a chronic infection of strongyloidiasis. Although he had no recent travel history apart from his time in Cuba as a young man, and now living in Miami Florida, his wide range of symptoms can be attributed to a disseminated strongyloidiasis infection. The rash, gastrointestinal upset, urinary retention, hemoptysis, eosinophilia, and SIADH can all be explained by disseminated strongyloidiasis infection in the setting of immunosuppression due to both his rituximab treatment for mantle cell lymphoma and corticosteroid treatment for COVID-19 upon previous discharge. 

After undergoing the first round of ivermectin treatment and receiving supportive medical care, the patient's hyponatremia, diarrhea, urinary retention, hemoptysis, and rash resolved.

## Discussion

Disseminated strongyloidiasis in immunocompromised patients presents unique challenges in terms of diagnosis and management. In this case study, we describe an 88-year-old Hispanic male with multiple comorbidities who developed disseminated strongyloidiasis, highlighting the importance of considering this parasitic infection in immunocompromised individuals with a diverse range of symptoms.

Immunosuppression increases the risk of *Strongyloides stercoralis* infection dissemination [1]. The motility rate of the parasite is significantly enhanced in immunosuppressed individuals, leading to a higher burden of infection and increased severity of symptoms.

The clinical presentation of disseminated strongyloidiasis can be diverse and nonspecific, making it challenging to recognize and diagnose. In our case, the patient exhibited symptoms, such as rash, gastrointestinal upset, urinary retention, and hyponatremia. Patients can also have chronic anemia and chronic eosinophilia. Although exceedingly rare, there have been previous reports of SIADH and urinary retention in patients with *Strongyloides *hyperinfection [4,7]. More importantly, widespread use of corticosteroids for COVID-19 treatment has led to *Strongyloides* reactivation and severe disease in patients from endemic areas [7,8]. These manifestations may share a common origin, thus further emphasizing the importance of considering strongyloidiasis as a potential underlying cause since undiagnosed disseminated strongyloidiasis infection can have a mortality rate as high as 90% [1]. 

Accurate diagnosis of strongyloidiasis can be challenging. Serial stool testing, blood agar plate culture, serologic testing, and real-time PCR can be utilized [1]. The treatment of disseminated strongyloidiasis typically involves antiparasitic medications, such as ivermectin, albendazole, or thiabendazole. Multidose therapy of ivermectin has shown to be efficacious in immunocompromised individuals [5]. 

This case study highlights the need for physicians to maintain a high level of suspicion for strongyloidiasis in immunocompromised individuals presenting with a diverse range of symptoms. Early recognition and diagnosis are essential to initiate appropriate treatment and prevent the dissemination of the infection.

## Conclusions

Despite its low prevalence within the United States, *Stronglyloides *infection proves to be a problematic diagnosis due to its insidious nature of progression making it very difficult to diagnose. Delayed diagnosis can be especially problematic with immunocompromised patients who are unable to mount an adequate immune response, which further predisposes them to manifestations of disseminated strongyloidiasis, such as cutaneous and respiratory symptoms in addition to the typical gastrointestinal presentation. It is imperative to have a degree of suspicion for a parasitic infection when patients present with non-specific symptoms and eosinophilia. These findings prompt the need for further workup to either rule out parasitic infection or ensure an early diagnosis. Early diagnosis allows for the prevention of prolonged and unnecessary hospital stays along with the initiation of adequate treatment, therefore reducing the risk of systemic infections and complications.
